# Editorial: Ion-channels in epilepsy

**DOI:** 10.3389/fneur.2023.1263539

**Published:** 2023-08-10

**Authors:** Enes Akyuz, Alberto Lazarowski, Carlos de Cabo, Leonid S. Godlevsky, Mohd. Farooq Shaikh, Zuleyha Doganyigit

**Affiliations:** ^1^Department of Pediatrics, University of Wisconsin-Madison, Madison, WI, United States; ^2^Department of Biophysics, University of Health Sciences, Istanbul, Türkiye; ^3^Department of Clinical Biochemistry, Hospital de Clinicas Jose de San Martin School of Pharmacy and Biochemistry, Institute for Research in Physiopathology and Clinical Biochemistry (INFIBIOC), University of Buenos Aires, Buenos Aires, Argentina; ^4^Neuropsychopharmacology Unit, Department of Research, Albacete General Hospital, Albacete, Spain; ^5^Odesa National Medical University, Ministry of Public Health of Ukraine, Odesa, Ukraine; ^6^School of Dentistry and Medical Sciences, Charles Sturt University, Orange, NSW, Australia; ^7^Neuropharmacology Research Laboratory, Jeffrey Cheah School of Medicine and Health Sciences, Monash University Malaysia, Bandar Sunway, Malaysia; ^8^Department of Histology and Embryology, Yozgat Bozok University, Yozgat, Türkiye

**Keywords:** ion channels, epilepsy, K channels, Na channels, seizure

The research title “Ion-channels in epilepsy” that we have prepared has accepted articles from important researchers in the field from countries such as the United States of America, Australia, Germany, China, and Spain. Four of these articles are original research, one of them is a brief research report article, and one is a review article. In the articles, the contribution of ion-channels to epilepsy has been examined. Summaries of these six articles are listed as follows.

Channelopathies are an emerging group of genetically determined neurologic disorders affecting the functionality of ion-channels. These disorders are characterized by a wide genetic and phenotypic heterogeneity. In the study by Koch et al. the effect of ion-channel mutations on neuronal firing was investigated by simulation. Through this, the importance of neuron type was discussed by predicting channelopathy in the K_V_1.1 channel encoded by the KCNA1 gene (Koch et al.). The simulations in the research have revealed the importance of personalized medicine approaches. Modeling approaches are also highlighted to provide insight into channelopathies quickly and efficiently.

In addition to the KCNA1 gene, changes in the KCNQ2 gene have also taken an important place in epilepsy studies and have become one of the regulators of neonatal brain excitability. In the ex vivo study conducted by Hou et al. it was shown that the ion-channel encoded by the *KCNQ2* gene regulates the population activity of GABAergic neurons in newborns (Hou et al.). This arrangement may be related to the loss of function in interneurons where the K_V_7.2 ion-channel is located, and it has been stated that stimulant transmission may contribute to the activity. In summary, the deletion of *KCNQ2*-encoded ion-channels from GABAergic cells leads to increased GABAergic interneuron population activity in the neonatal forebrain. The study can regulate both immature excitatory neuron networks and GABAergic interneuron networks of ion-channels encoded by *KCNQ2* genes. This information predicts that *KCNQ2* can modulate the activity of both glutamatergic and GABAergic cells.

Another type of epilepsy in which genes encoding potassium channels are involved is temporal lobe epilepsy (TLE). The fact that this type of epilepsy is the most common and drug-resistant has attracted the attention of researchers in recent years. Experimental validation accompanying the bioinformatics analyzes by Zhang et al. highlighted four potassium channel genes: *KCNA1, KCNA2, KCNJ11*, and *KCNS1*. The expression of these genes in the brain tissues of mice induced in the epilepsy model agrees with human data (Zhang et al.). This agreement showed the importance of bioinformatics analyzes and highlighted the role of potassium channel genes in TLE. The four resulting genes may indirectly affect the balance between inhibitory and excitatory neurons through the respective potassium channel down-regulation mechanism. Therapeutic strategies targeting these genes may give direction to future research.

*KCNC2* gene variants encoding the K_V_3.2 channel are known to be associated with epilepsy. In their functional studies, Seiffert et al. showed that the effect of this gene on four variants is uncertain. This suggests that the therapeutic effect of valproic acid can be explained by other new mechanisms.

The balance between the activation of Na+/K+/Cl- (NKCC1) and K+/Cl- (KCC2) proteins, which are co-transporters supporting the ionic movements of ion-channels and direct the movement of Cl ions in and out, respectively, is important in epilepsy. Bonet-Fernandez et al. contributed to the examination of this balance by investigating the contribution of NKCC1 to GABA neurotransmission in the epileptic seizure model. This study reveals that the NKCC1 co-transporter is sensitive to seizures in brain regions and promotes the upregulation of NKCC1 as a contributing factor to the dysregulation of GABA neurotransmission (Bonet-Fernandez et al.). NKCC1 studies need to be increased to discover and improve therapeutic approaches.

The contribution of the dysfunction in the Na_V_1.1 ion-channel encoded by the *SCNA1* gene to epilepsy was discussed by Bryson and Petrou in the review article. This discussion emphasized that genetic and cellular disease mechanisms are related and that cellular examination with a microcircuit approach is required in all *SCN1A*-related epilepsy disorders (Bryson and Petrou).

As the editorial team, we would like to thank all the contributing authors. These articles will breathe new life into research on ion-channels in epilepsy and will encourage other researchers. Every new piece of research represents a step toward a better understanding of epileptogenesis and improving epilepsy treatment.

## Author contributions

EA took the initiative for the editorial write-up. AL, CC, LG, MS, and ZD contributed to revising and proofreading. All authors contributed to the article and approved the submitted version.

